# Transcriptome analysis between invasive Pomacea canaliculata and indigenous Cipangopaludina cahayensis reveals genomic divergence and diagnostic microsatellite/SSR markers

**DOI:** 10.1186/s12863-015-0175-2

**Published:** 2015-02-11

**Authors:** Xidong Mu, Guangyuan Hou, Hongmei Song, Peng Xu, Du Luo, Dangen Gu, Meng Xu, Jianren Luo, Jiaen Zhang, Yinchan Hu

**Affiliations:** Pearl River Fisheries Research Institute, Chinese Academy of Fishery Sciences, Key Laboratory of Tropical&Subtropical Fishery Resource Application&Cultivation, Ministry of Agriculture, Guangzhou, 510380 China; Center for Applied Aquatic Genomics, Chinese Academy of Fishery Sciences, Beijing, 100141 China; Department of Ecology, College of Agriculture, South China Agricultural University, Key Laboratory of Ecological Agriculture, Guangzhou, 510642 China

**Keywords:** Biological invasion, Pomacea canaliculata, Cipangopaludina cahayensis, EST-SSR, Transcriptome

## Abstract

**Background:**

Pomacea canaliculata is an important invasive species worldwide. However, little is known about the molecular mechanisms behind species displacement, adaptational abilities, and pesticide resistance, partly because of the lack of genomic information that is available for this species. Here, the transcriptome sequences for the invasive golden apple snail *P. canaliculata* and the native mudsnail *Cipangopaludina cahayensis* were obtained by next-generation-sequencing and used to compare genomic divergence and identify molecular markers.

**Results:**

More than 46 million high quality sequencing reads were generated from *P. canaliculata* and *C. cahayensis* using Illumina paired-end sequencing technology. Our analysis indicated that 11,312 unigenes from *P. canaliculata* and *C. cahayensis* showed significant similarities to known proteins families, among which a total of 4,320 specific protein families were identified. KEGG pathway enrichment was analyzed for the unique unigenes with 17 pathways (*p*-value < 10^−5^) in *P. canaliculata* relating predominantly to lysosomes and vitamin digestion and absorption, and with 12 identified in *C. cahayensis*, including cancer and toxoplasmosis pathways, respectively. Our analysis also indicated that the comparatively high number of P450 genes in the *P. canaliculata* transcriptome may be associated with the pesticide resistance in this species*.* Additionally, 16,717 simple sequence repeats derived from expressed sequence tags (EST-SSRs) were identified from the 14,722 unigenes in *P. canaliculata* and 100 of them were examined by PCR, revealing a species-specific molecular marker that could distinguish between the morphologically similar *P. canaliculata* and *C. cahayensis* snails.

**Conclusions:**

Here, we present the genomic resources of *P. canaliculata* and *C. cahayensis*. Differentially expressed genes in the transcriptome of *P. canaliculata* compared with *C. cahayensis* corresponded to critical metabolic pathways, and genes specifically related to environmental stress response were detected. The CYP4 family of P450 cytochromes that may be important factors in pesticide metabolism in *P. canaliculata* was identified. Overall, these findings will provide valuable genetic data for the further characterization of the molecular mechanisms that support the invasive and adaptive abilities of *P. canaliculata*.

**Electronic supplementary material:**

The online version of this article (doi:10.1186/s12863-015-0175-2) contains supplementary material, which is available to authorized users.

## Background

Biologically invasive species are one of the major threats to global biodiversity, and they can cause substantial economic losses as well as pose a public health risk [1-8]. The golden apple snail (*Pomacea canaliculata*) is native to South America and is beginning to emerge worldwide, among others China. It has become a highly damaging invasive species, affecting agriculture and fisheries, as well as pubilc heatlth [9-14]. The snail was first introduced to Zhongshan (Guangdong Province, China) as a human food source or aquarium pet [15]. It adapted quickly and is now found at least 11 provinces in southern China [16]. Currently, *P. canaliculata* has invaded local habitats, including rice fields and ponds, causing severe crop damage and substantial ecological destruction such as the destruction of aquatic product resources [9,17,18] and the displacement of the native mudsnail *Cipangopaludina cahayensis*. In addition, *P. canaliculata* serves as a major intermediate host for the nematode *Angiostrongylus cantonensis*, which has led to the emergence of human eosinophilic meningitis in China [16,19].

Genetic divergence between the alien and native species may play an important role in the highly adaptive nature of *P. canaliculata*. However, few genomic resources are available for *P. canaliculata* and *C. cahayensis*, and this lack of information has hindered the understanding of possible molecular mechanisms [20]. Previous studies using mitochondrial DNA have provided insights into the continental expansion and molecular phylogeny of *P. canaliculata* [12,13,18,21-24], but any genomic factors pertaining to competition and displacement are still unknown.

Recently, next generation sequencing technologies have revolutionized the fields of genomics and transcriptomics, providing an opportunity for the rapid and cost-effective generation of genome-scale data [25]. These technologies have been applied successfully in many invasive species, including *Bemisia tabaci* [26,27], *Anguillicola crassus* [28], *Aedes aegypti* [29] and *Mytilus galloprovincialis* [30]. In the present study, we sequenced and assembled the transcriptome of the native *C. cahayensis* from mainland China and the invasive *P. canaliculata* using *de novo* sequence assembly. Transcriptome divergence between the native and invasive species was examined to identify important candidate genes related to competitiveness, resistance to environmental stress, and invasive potential. This approach enabled the prediction of expressed sequence tag-simple sequence repeat (EST-SSR) markers to facilitate gene mapping and genetic variation analysis in *P. canaliculata*.

## Result and discussion

### Sequencing data and *de novo* assembly

**Figure 1:**
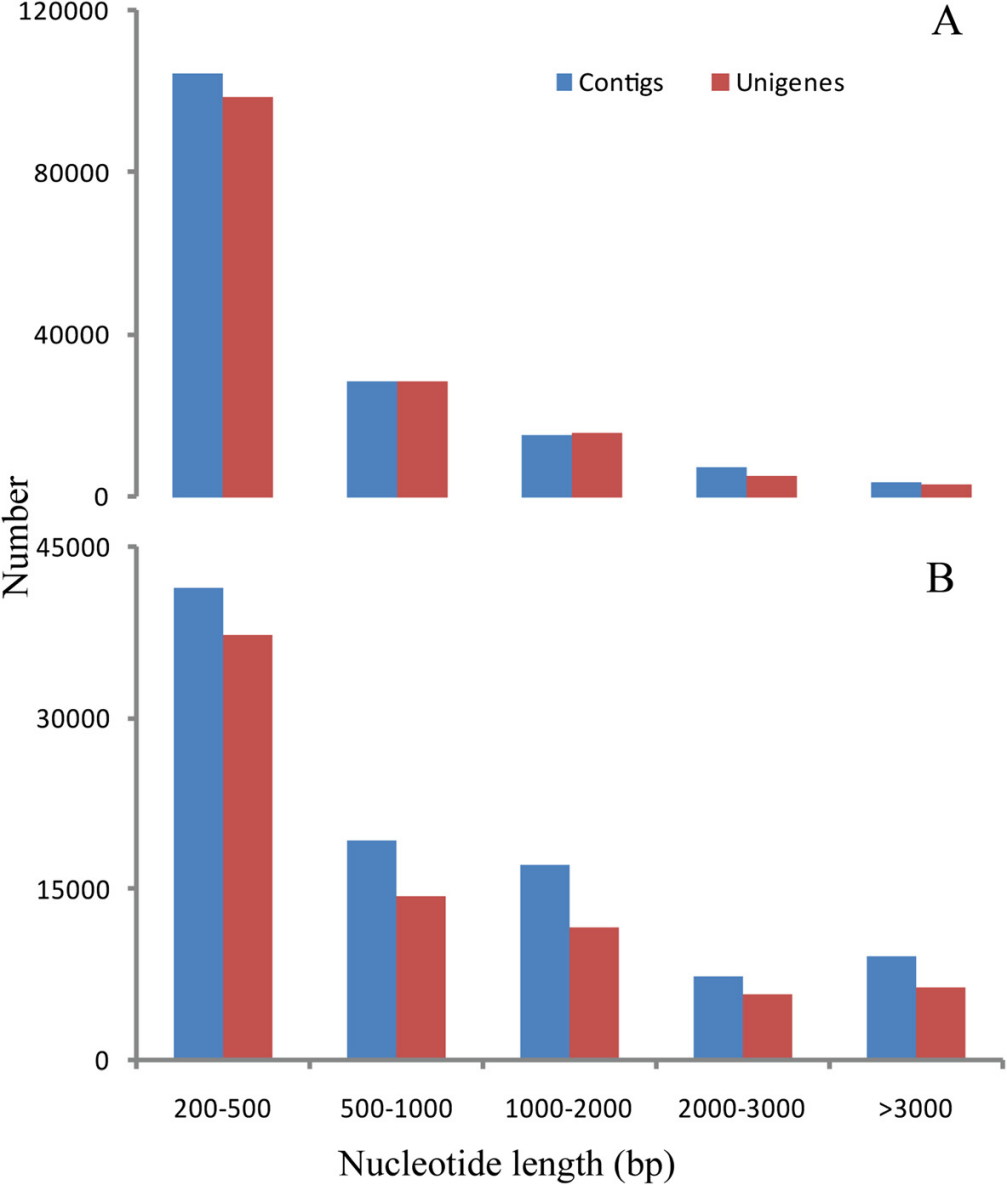
**Assessment of transcriptome assembly quality of Cipangopaludina cahayensis (A) and Pomacea canaliculata (B).**

Using Illumina paired-end sequencing technology, the transcriptome sequencing produced 65,198,546 reads with a total length of 6.5 Gb for *C. cahayensis*, which generated 161,941 contigs and 151,518 unigenes (Table 1). For *P. canaliculata*, 94,808,488 reads were obtained, and 94,518 contigs and 76,082 unigenes were generated (Table 1). Using the SOAP *de novo* assembly program, high quality reads were assembled into 160,256 contigs longer than 200 bp, with a mean length of 1,080 bp and a N50 of 1,004 bp for the native *C. cahayensis*. For *P. canaliculata,* 94,518 contigs longer than 200 bp, with a mean length of 916 bp and a N50 of 1,854 bp were generated. In *C. cahayensis*, the lengths of 104,713 (65.34%) of the contigs ranged from 200 to 500 bp, 28,918 (18.04%) contigs ranged from 500 to 1,000 bp, and 15,191 (9.50%) contigs ranged from 1000 to 2,000 bp; the remaining contigs were longer than 2,000 bp (Figure 1). In *P. canaliculata*, the lengths of 41,544 (43.95%) of the contigs ranged from 200 to 500 bp, 19,289 (20.41%) contigs ranged from 500 to 1000 bp, and 17,619 (18.16%) contigs ranged from 1000 to 2,000 bp; the remaining contigs were longer than 2,000 bp. The related data were submitted to the NCBI data under accession numbers: SRA191276 (*P. canaliculata*) and SRA192725 (*C. cahayensis*).

**Table 1:** **Transcriptome summary for indigenous** ***Cipangopaludina cahayensis*** **and** ***Pomacea canaliculata***

	***Cipangopaludina cahayensis***	**Pomacea canaliculata**
Total number of reads	65,198,546	94,808,488
Total base pair (bp)	6,519,854,600	3,507,914,056
Total number of contigs	161,941	94,518
Mean length of contigs (bp)	1,080	916
Total number of Unigenes	151,518	76,082
Mean length of Unigenes (bp)	1,004	1,854

### Functional annotation

**Figure 2:**
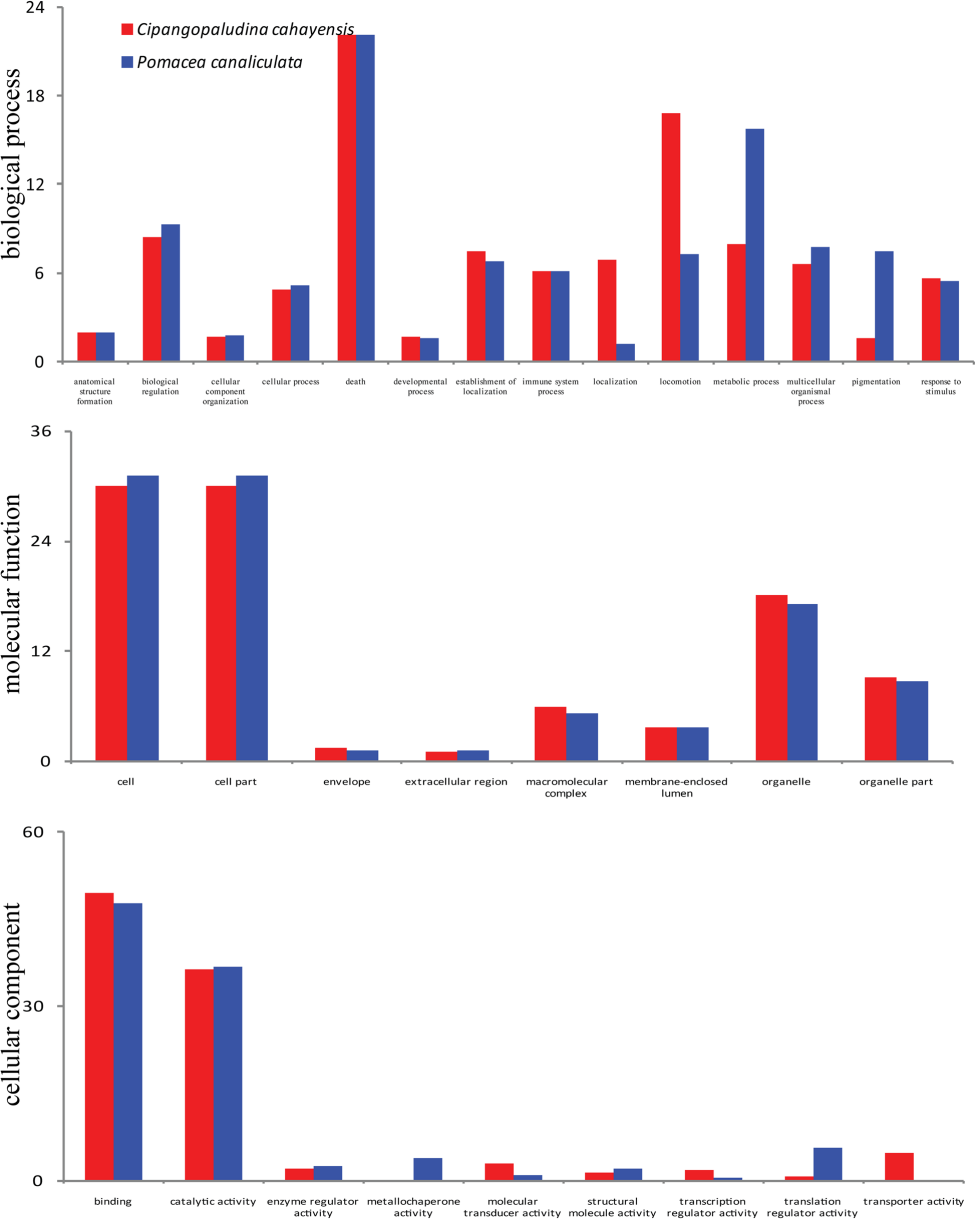
**Comparing functional annotations of contigs between Cipangopaludina cahayensis (red) and invasive Pomacea canaliculata (blue) transcriptome.** The distribution of gene ontology (GO) terms is given for each of each of the three main GO categories (biological process, molecular function, and cellular component).

To annotate the *C. cahayensis* and *P. canaliculata* sequences, searches were conducted against the NCBI non-redundant protein (Nr) database, the Swiss-Prot protein database, Cluster of Orthologous Groups (COG), and Kyoto Encyclopedia of Genes and Genomes (KEGG) database using BLASTX (*E*-value ≤ 1 × 10^−5^). The alignment results were used to predict unigene transcriptional orientations and coding regions. Gene ontology (GO) terms were assigned to the annotated sequences and 14,864 sequences from *C. cahayensis* and 56,300 sequences from *P. canaliculata* were categorized into the three GO categories, biological process, cellular component, and molecular function (Figure 2). We found that the distribution and percentages of the assigned gene functions were similar in both species. In the biological process category, death (22.1%) was prominent, while in the molecular function category, cell (30%–31%) and cell part (30%–31%) were prominently represented. In the cellular component category, binding (47.8%–49%) was predominant, followed by catalytic activity (36%). Overall, the transcriptome sequencing yielded a great number of unique genes in the two species, in agreement with similar results reported in other species [20]. Several differences were noted between the two species, with more genes noted in *P. canaliculata* (56,300 genes) compared with in *C. cahayensis* (14,864 genes). Furthermore, the percentage of genes annotated as metabolic process/pigmentation under the biological process category was higher in *P. canaliculata* (15.7%/7.46%) compared with *C. cahayensis* (7.93%/1.6%), implying a possible relation to various environmental stressors. Moreover, the percentage of genes annotated as metallochaperone activity and translation regulator activity under the cellular component category was much higher in *P. canaliculata* compared with *C. cahayensis.* These results indicated that *P. canaliculata* might contain additional genes that are able to confer high competitiveness or strong resistance to envrionmental stress compared to *C. cahayensis.*

**Figure 3:**
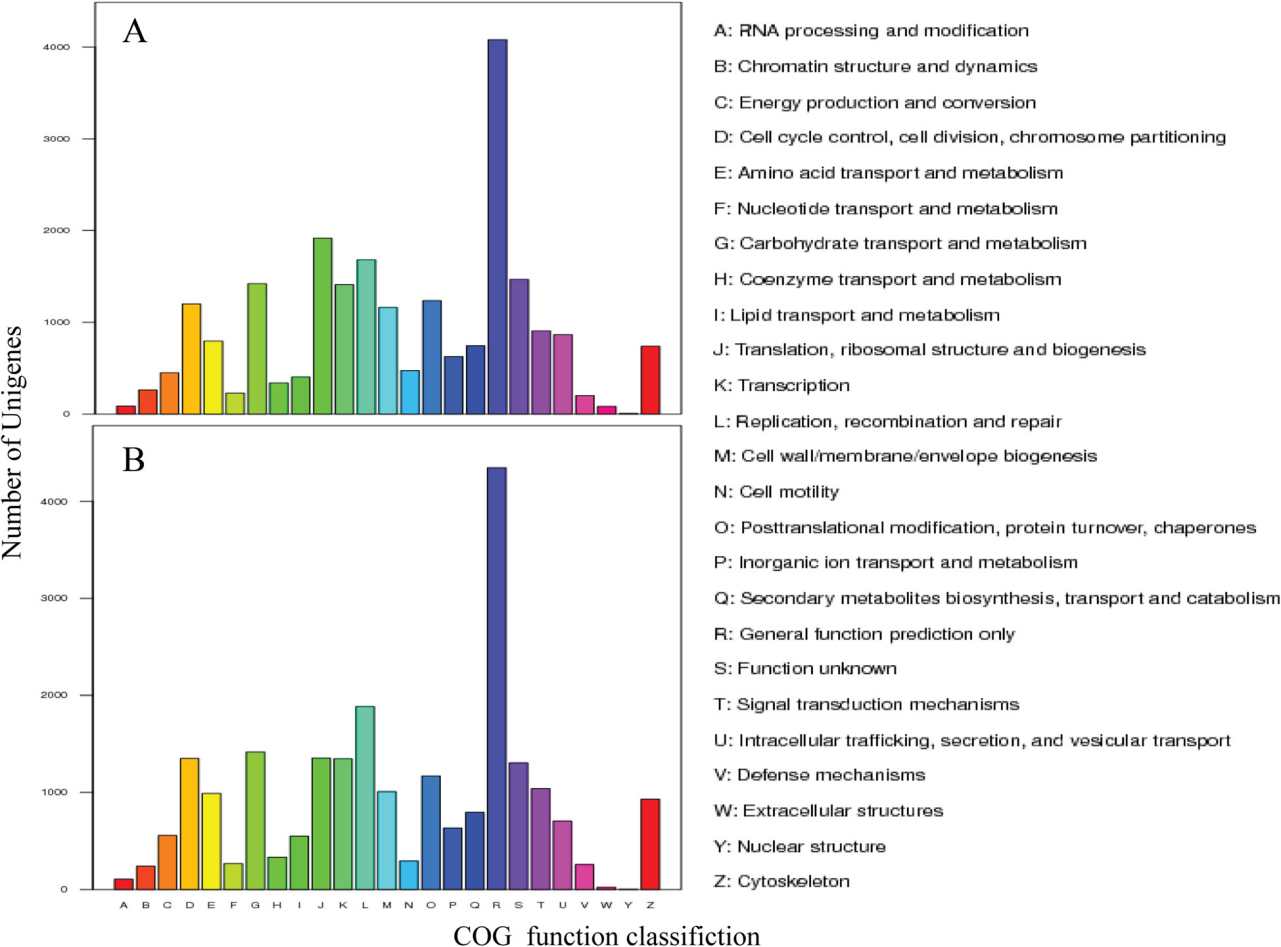
**Clusters of orthologous group (COG) classifications for Cipangopaludina cahayensis (A) and Pomacea canaliculata (B) transcriptome.** All unigenes were aligned to COG database to predict and classify possible functions.

Furthermore, all of the *C. cahayensis* and *P. canaliculata* unigenes were subjected to functional prediction and classification using the COG database. The unigenes were assigned to 25 COG categories (Figure 3), among which “general function prediction” represented the largest group (4,081 (17.9%) genes for *C. cahayensis*; 4,346 (19%) genes for *P. canaliculata*). For *C. cahayensis*, the next most represented category was translation, ribosomal structure and biogenesis (1915 (8.41%) genes), while for *P. canaliculata*, replication, recombination and repair (1,883 (8.23%) genes,) was the next most represented category.

To identify differentially regulated biological pathways between *C. cahayensis* and *P. canaliculata*, the annotated unigenes were mapped to reference pathways in the KEGG database [31]. We found that 13,351 *C. cahayensis* unigenes mapped to 276 pathways and 13,808 *P. canaliculata* genes mapped to 240 pathways, with different pathway associations between the two species. In *C. cahayensis*, the largest number of genes included cancer (577 (4.32%) genes; pathway: ko05200), focal adhesion (496 (3.72%) genes; pathway: ko04510), ubiquitin mediated proteolysis (427 (3.2%) genes; pathway: ko04120), and Huntington’s disease (333 (2.49%) genes; pathway: ko05016). In *P. canaliculata*, the predominant pathways were metabolic (2241 (16.23%) genes; pathway: ko01100), cancer (530 (3.84%) genes; pathway: ko05200), focal adhesion (415 (3.01%) genes; pathway: ko04510) and Huntington’s disease (348 (2.52%) genes; pathway: ko05016). Collectively, these transcriptome sequences and pathway annotations provide an essential resource for further screening and expression analysis of candidate genes related to the invasive abilities of *P. canaliculata*.

### Analysis of protein families and genes

**Figure 4:**
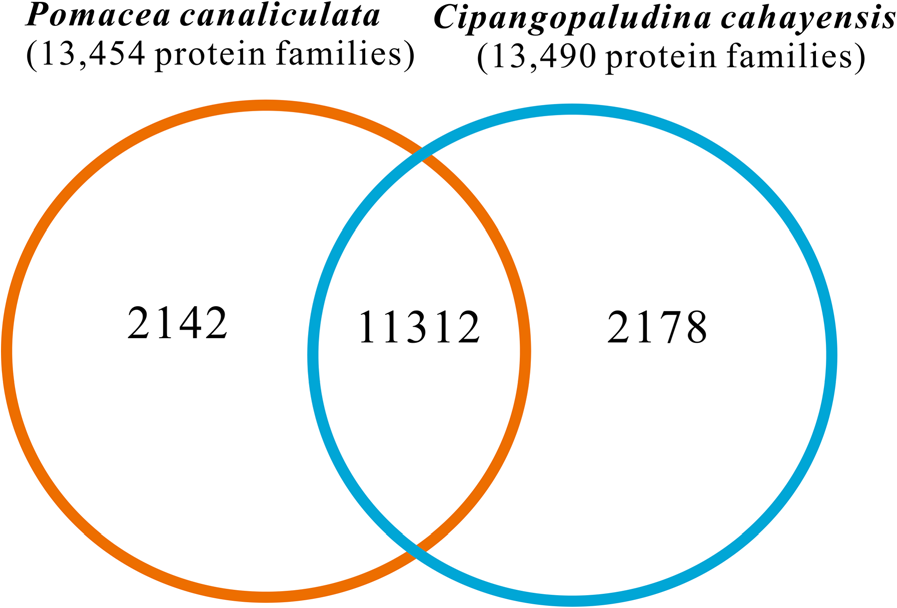
**Protein families from the transcriptomes of Cipangopaludina cahayensis and Pomacea canaliculata.** Protein families were identified for all the translated genes of the two transcriptomes using Blastp and a Markov Cluster algorithm (MCL), with the total number of protein families belonging to each category listed in the figure for the 11,312 protein families belonging to the two transcriptomes.

A total of 15,632 protein families were identified based on sequence similarities (Figure 4); 13,490 families for *C. cahayensis* and 13,453 families for *P. canaliculata*. When the transcriptomes of the two species were compared, a total of 11,312 protein families were found to be conserved between the *C. cahayensis* and *P. canaliculata* transcriptomes, and 2142 and 2178 families for *P. canaliculata* and *C. cahayensis*, respectively, were found to be differentially expressed. Some of the differentially expressed proteins may be responsible for the unique features of each of these species. An enriched analysis of the GO terms assigned to the 11,312 conserved protein families, identified 12 protein families that were significantly enriched (Table 2), including RNA transport (380 (2.6%) genes), spliceosome (383 (2.62%) genes), and endoplasmic reticulum protein processing (358 (2.45%) genes), which are related to protein transportation and metabolism. The finding that GO terms related to protein transportation and metabolism were enriched is inconsistent with the results reported for other invasive species such as *Bemisia tabaci* [32], possibly suggesting the critical roles of these pathways in these two species. We identified a total of 12 protein families (*p*-value < 10^−5^) encoded by the differentially expressed genes in *C. cahayensis* (Table 3), including those assigned to pathways pertaining to cancer (97 (6.92%) genes), toxoplasmosis (87 (6.21%) genes), and apoptosis (71 (5.06%) genes). In *P. canaliculata*, we identified a total of 17 protein families (*p*-value < 10^−5^) encoded by the differentially expressed genes, including those assigned to pathways pertaining to lysosomes (84 (4.02%) genes), vitamin digestion and absorption (71 (3.4%) genes), ECM-receptor interaction (57 (2.73%) genes), and metabolism of xenobiotics by cytochrome P450 (49 (2.35%) genes). We used reads per kilobase per million mapped reads (RPKM) to analyze the expression levels of *P. canaliculata* genes and identified 20 annotated genes with very high expression levels (RPKM > 2000), which were predicted to be involved in cell and protein structure (ferritin [Swiss-Prot: C7TNT3] and augerpeptide hhe53 [Swiss-Prot: P0CI21]) and ribosomes (60S ribosomal proteins and 40S ribosomal protein S8) (Table 4).

**Table 2:** **Statistically common enriched Gene Ontology (GO) terms between** ***Cipangopaludina cahayensis*** **and** ***Pomacea canaliculata*** **for the 11,312 protein families**

**KO term**	**No. of DEGs**	**No. of genes**	**p-value**	**Pathways**
ko03050	87 ((0.6%))	104 (0.38%)	1.177307e-10	proteasome
ko04141	358 (2.45%)	533 (1.96%)	1.450501e-10	protein processing in endoplasmic reticulum
ko03013	380 (2.6%)	571 (2.1%)	2.417814e-10	RNA transport
ko00190	222 (1.52%)	315 (1.16%)	7.983256e-10	oxidative phosphorylation
ko00020	111 (0.76%)	148 (0.54%)	7.634897e-08	citrate cycle (TCA cycle)
ko00010	143 (0.98%)	199 (0.73%)	1.168776e-07	Glycolysis/Gluconeogenesis
ko04130	65 (0.45%)	80 (0.29%)	2.460867e-07	SNARE interactions in vesicular transport
ko00280	131 (0.9%)	183 (0.67%)	5.546992e-07	Valine, leucine and isoleucine degradation
ko03040	383 (2.62%)	609 (2.24%)	2.553937e-06	spliceosome
ko00030	69 (0.47%)	89 (0.33%)	2.854414e-06	pentose phosphate pathway
ko04910	287 (1.97%)	453 (1.67%)	1.953777e-05	insulin signaling pathway
ko04380	155 (1.06%)	232 (0.85%)	3.401093e-05	osteoclast differentiation

**Table 3:** **Statistically unique protein families in** ***Cipangopaludina cahayensis*** **and** ***Pomacea canaliculata***

**KO term**	No. of DEGs*	No. of genes**	**p-value**	**Pathway**
Cipangopaludina cahayensis				
ko05145	87 (6.21%)	316 (2.37%)	8.262866e-18	toxoplasmosis
ko04210	71 (5.06%)	251 (1.88%)	2.041735e-15	apoptosis
ko05222	64 (4.56%)	264 (1.98%)	8.751653e-11	small cell lung cancer
ko05144	23 (1.64%)	55 (0.41%)	1.741389e-09	malaria
ko04621	47 (3.35%)	190 (1.42%)	1.417618e-08	NOD-like receptor signaling pathway
ko05014	29 (2.07%)	102 (0.76%)	3.722068e-07	amyotrophic lateral sclerosis (ALS)
ko05200	97 (6.92%)	577 (4.32%)	1.527724e-06	pathways in cancer
ko05146	39 (2.78%)	170 (1.27%)	1.852183e-06	amoebiasis
ko00590	29 (2.07%)	119 (0.89%)	1.106722e-05	arachidonic acid metabolism
ko05416	35 (2.5%)	158 (1.18%)	1.392634e-05	viral myocarditis
ko05210	27 (1.93%)	116 (0.87%)	5.248618e-05	colorectal cancer
ko05323	20 (1.43%)	76 (0.57%)	7.901248e-05	rheumatoid arthritis
Pomacea canaliculata				
ko00940	22 (1.05%)	23 (0.17%)	1.61937e-17	Phenylpropanoid biosynthesis
ko04977	71 (3.4%)	172 (1.25%)	7.427869e-17	vitamin digestion and absorption
ko00140	52 (2.49%)	120 (0.87%)	1.004048e-13	Steroid hormone biosynthesis
ko04512	57 (2.73%)	158 (1.14%)	5.811497e-11	ECM-receptor interaction
ko00830	49 (2.35%)	126 (0.91%)	6.057395e-11	Retinol metabolism
ko00980	49 (2.35%)	129 (0.93%)	1.610139e-10	Metabolism of xenobiotics by cytochrome P450
ko00130	24 (1.15%)	41 (0.3%)	2.008634e-10	Ubiquinone and other terpenoid-quinone biosynthesis
ko00591	45 (2.15%)	116 (0.84%)	4.002728e-10	linoleic acid metabolism
ko00533	22 (1.05%)	38 (0.28%)	1.543998e-09	glycosaminoglycan biosynthesis - keratan sulfate
ko00740	21 (1.01%)	37 (0.27%)	5.989671e-09	riboflavin metabolism
ko00360	26 (1.24%)	54 (0.39%)	1.007722e-08	Phenylalanine metabolism
ko00982	47 (2.25%)	142 (1.03%)	6.201926e-08	drug metabolism-cytochrome P450
ko00627	41 (1.96%)	119 (0.86%)	1.229762e-07	aminobenzoate degradation
ko04142	84 (4.02%)	321 (2.32%)	1.553304e-07	lysosome
ko00590	45 (2.15%)	138 (1%)	1.921892e-07	arachidonic acid metabolism
ko00983	47 (2.25%)	147 (1.06%)	2.012819e-07	drug metabolism-other enzymes
ko02020	16 (0.77%)	31 (0.22%)	2.208469e-06	two-component system

*The number of differentially expressed genes (DEGs) that belong to a KEGG pathway.

**The total number of orthologous genes that belong to a KEGG pathway.

**Table 4:** **Highly expressed genes in the transcriptome of** ***Pomacea canaliculata***

**Gene ID**	Number of reads*	RPKM**	**Swissprot annotation**	**E-value**
Unigene0034597	370469	7963.2	Ferritin	2.00E-79
Unigene0070417	178665	6508.7	Temptin	4.00E-22
Unigene0099572	1758831	6234.6	Auger peptide hhe53	1.00E-11
Unigene0095431	309172	5711.9	Cysteine-rich secretory protein Mr30	6.00E-50
Unigene0102121	48348	5217.9	Polyubiquitin	7.00E-39
Unigene0069599	333123	4748.7	Elongation factor 1-alpha, somatic form	0
Unigene0122375	169714	4112.1	Fibrinogen C domain-containing protein 1-B	1.00E-53
Unigene0115512	780688	4335.5	Paramyosin	0
Unigene0087254	227698	4289.3	Metalloproteinase inhibitor 3	7.00E-07
Unigene0114631	361080	4118.2	Actin, adductor muscle	0
Unigene0069690	284495	4020.1	Tubulin beta chain	0
Unigene0121686	60035	3534.1	60S ribosomal protein L36	2.00E-31
Unigene0006316	82422	2973.3	40S ribosomal protein S8	1.00E-89
Unigene0102783	157410	2712.7	60S ribosomal protein L5	1.00E-123
Unigene0033792	48918	2226.8	60S ribosomal protein L24	4.00E-57
Unigene0099167	208322	2530.9	Myosin, essential light chain, adductor muscle	1.00E-47
Unigene0083872	75749	2346.9	60S ribosomal protein L44	3.00E-47
Unigene0099241	78798	2247.8	60S ribosomal protein L7a	2.00E-113
Unigene0034297	55506	2045.0	60S ribosomal protein L23a	1.00E-57
Unigene0123696	49155	2011.4	Ubiquitin-60S ribosomal protein L40	7.00E-68

*The total number of reads mapped to each gene.

**Gene expression levels were determined by calculating the number of reads for each gene and then normalizing to RPKM.

**Figure 5:**
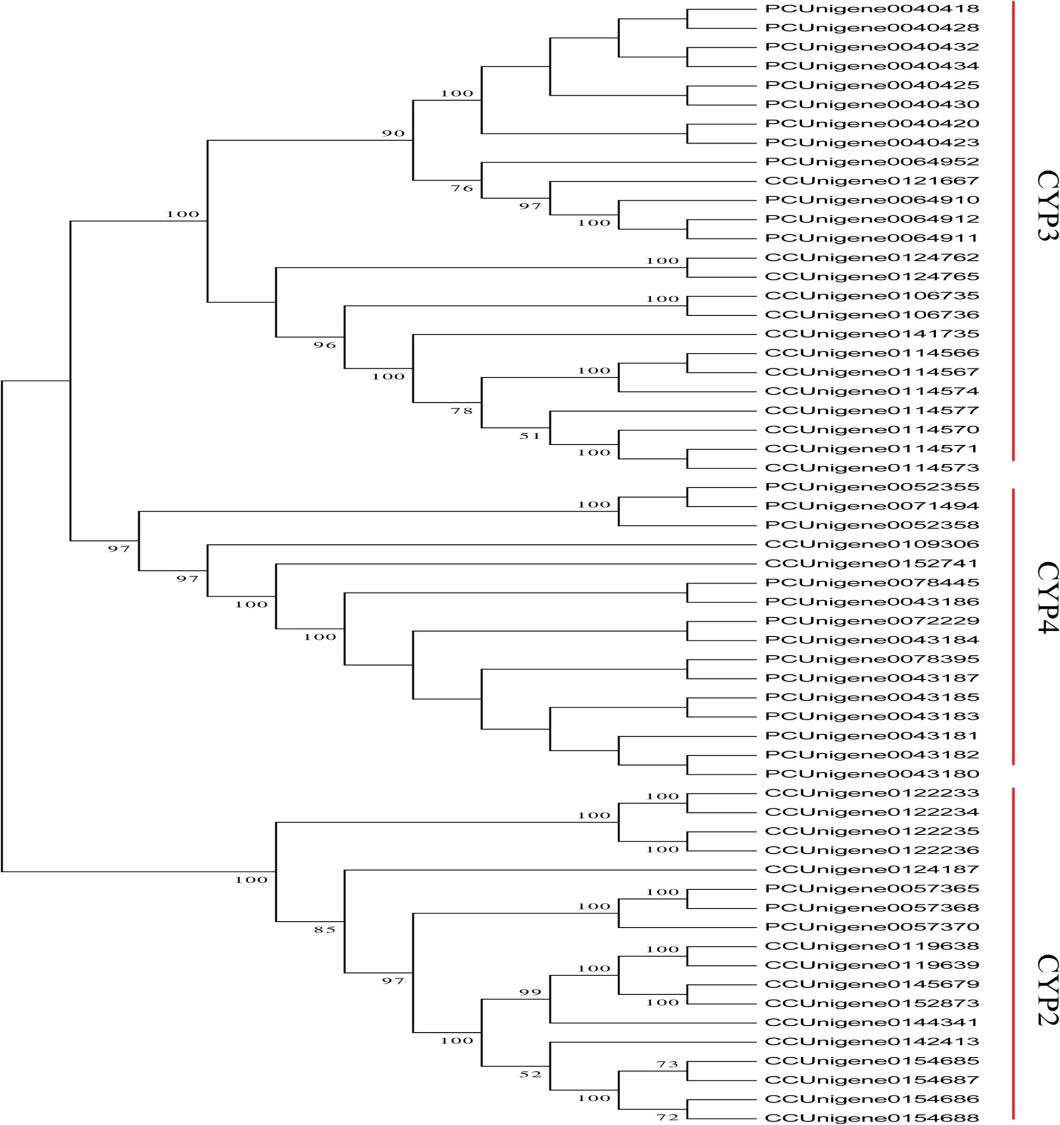
**Neighbor-joining phylogenetic analysis of cytochrome P450 from Cipangopaludina cahayensis (CC) and Pomacea canaliculata (PC).** CYP represent cytochrome P450.

*P. canaliculata* has become an important pest in China and has exhibitied resistance to pesticides such as metaldehyde and niclosamide ethanolamine salt [33-35]; however, the molecular mechanisms underlying this resistance are still unclear. To detect unique resistance-related sequences, the unigenes were edited manually to remove redundant and overlying short sequences and the edited sequences were then used to identify genes encoding proteins related to the metabolism of pesticides. We identified P450 cytochromes (CYPs), a major family of enzymes involved in detoxification and metabolism, as potential major detoxification component proteins [36-38]. Previous studies have reported a correlation between increased exposure to metabolic neurotoxic pesticides and over-expression of P450 genes in many pest species [39-46]. In our study, 210 P450-related sequences were identified in *P. canaliculata* and only 159 were found in *C. cahayensis*, indicating that the number of P450 genes may be one of the contributory factors to pesticides resistance in *P. canaliculata*. While the number of P450 genes detected is not necessarily related to gene expression levels, an increased gene number of genes may increase metabolic enzyme detoxification activity, and contribute to the development of a progressive resistance in *P. canaliculata*. These findings will enhance the understanding of pesticide metabolism and help in the development of effective treatments for invasive species. To investigate the relationship between the P450 sequences from both species a phylogenetic tree was constructed using the neighbor joining (NJ) method in conjunction with bit-score values. Sixty of the sequences showed high homology and were classified into the CYP2, CYP3, and CYP4 families based on their similarity to sequences in the Nr database. These sequences clustered into three clades in the phylogentic tree that corresponded to the same three P450 families (Figure 5). We found a high concentration of *P. canaliculata* genes in the CYP4 family, possibly implying that these genes played important roles in the metabolism of pesticides in this invasive species. While these finding are insightful, they need to be examined further using RACE technology and RT-PCR before they can be accepted.

### Detection of intraspecific genetic variation

**Figure 6:**
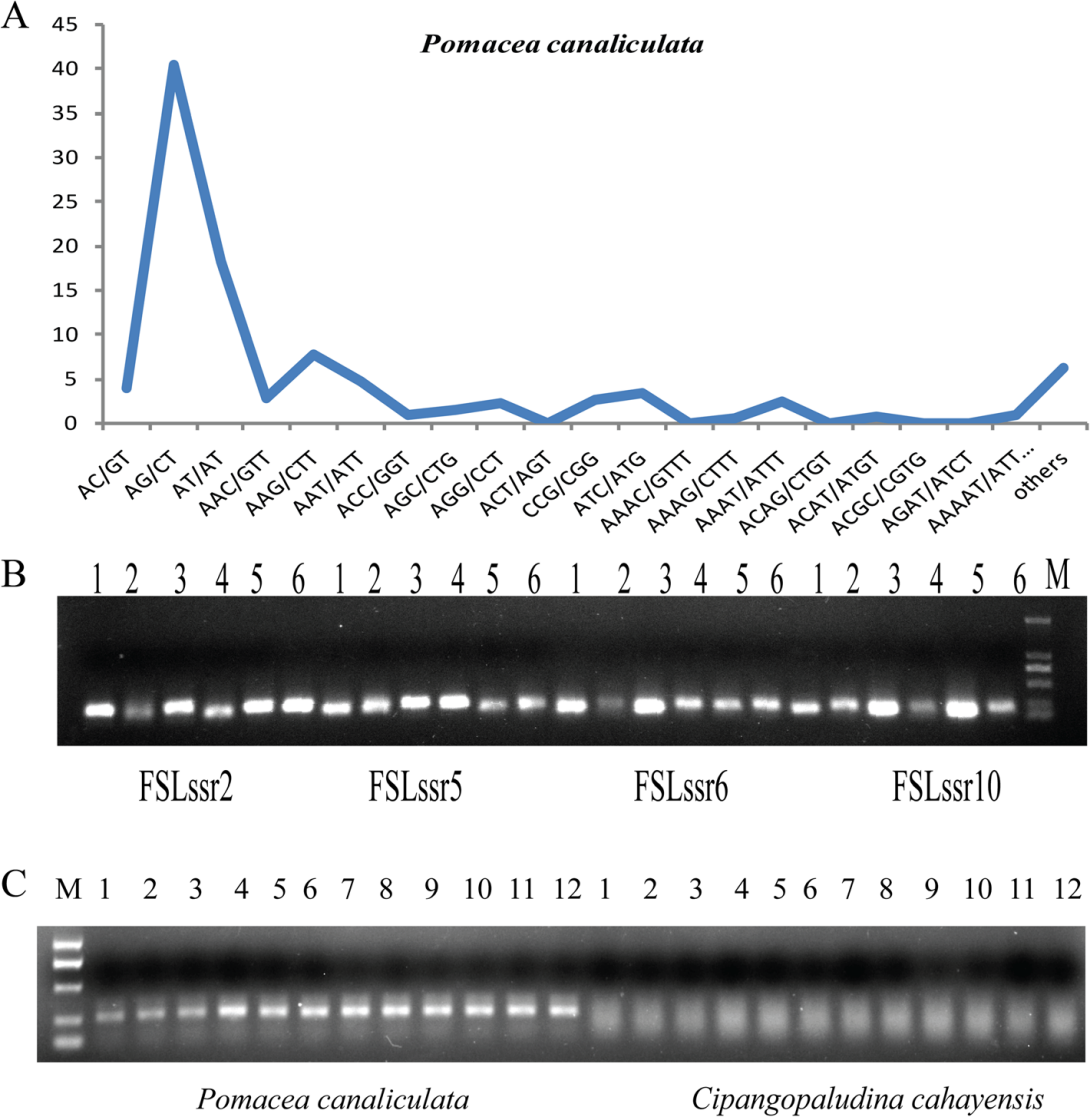
**Frequencies and polymorphisms of classified SSR repeat types and molecular characterization of Pomacea canaliculata. (A)**: The graph shows the frequency of each repeat motif classified, considering the sum of the frequencies for complementary sequences (for example, the sum of frequencies for the dinucleotides AC and its complementary GT). **(B)** Polymorphism and validation of a subset of the microsatellite primer pairs for six P. canaliculata samples by agarose-gel profiling. 1–6 represent GZ1, GZ2, HN1, HN2, SG1, and SG2, respectively. **(C)** The SSR primer (FSLssr64) for species-specific identification between *P. canaliculata* and *C. cahayensis.*

EST-SSRs serve as effective molecular markers for genetic mapping, comparative genomics and population genetic analysis in many invasive species. Characterization of EST-SSRs may enable breakthroughs in the detection of cryptic species, aid in defining the number and location of establishment events, and help trace the routes of alien species as they spread into new regions [47-51]. Compared with traditional methods, EST-SSRs are more transferable and advantageous than random genomic SSRs, enabling improved genetic studies related to population genetics [52]. Unitl now, only a few SSRs have been identified in *P. canaliculata* [20,53], which has hampered marker applications in this species. To further understand the invasive and adaptive mechanism in *P. canaliculata*, six *P. canaliculata* samples were collected from three. invasive regions/habitats in mainland China and examined for polymorphisms. A total of 16,717 potential SSRs were identified. As shown in Table 5, the di-nucleotide repeats were the most abundant (10,554, 63.1%), followed by tri-(4,480, 26.8%), tetra- (1,021, 6.10%), hexa-(341, 2.0%), and penta-nucleotide (321, 1.9%) repeats. The most abundant repeat combination was AG/CT (40.4%), followed by AT/AT (18.3%), AAG/CTT (7.8%), AAT/ATT (4.7%), AC/GT (4.0%) and ATC/ATG (3.4%) (Figure 6A). Based on the SSR-containing sequences, 8,428 SSR primers were developed and 100 SSRs (Additional file 1: Table S1) were selected to design EST-SSR primers based on the information (name and longer length of gene identified). Of the 100 SSRs examined by PCR amplification, 26 (26.0%) PCR products exhibited more than one band, which may have resulted from high heterozygosity, while the others SSRs generated bands of the expected length. In total, 143 amplicons were detected from the 100 primer pairs. The number of amplicons per primer pair ranged from one to three, with an average of 1.43 (Figure 6B). To estimate EST-SSR marker novelty, the amplicons were evaluated against previously reported *P. canaliculata* markers [20,53]. We found that the 100 EST-SSR markers had not been reported previously. Thus, other EST-SSR primers can be designed from the 8,428 identified EST-SSR to contribute further to the characterization of the invasive and adaptive processes. *P. canaliculata* and *C. cahayensis* have very similar morphological features, especially at the immature stages, which makes early identification difficult. Therefore, a molecular means for the identification and characterization of these two species is essential. Using the *P. canaliculata* SSR primers, we identified a unique amplicon (FSLssr64; Additional file 1: Table S1) that was present in *P. canaliculata* but absent in *C. cahayensis* (Figure 6C). Thus, FSLssr64 could serve as a species-specific molecular marker to distinguish these two species and aid in the prevention and detection of invasive *P. canaliculata* in different regions.

**Table 5:** **Summary of EST-SSRs identified in the** ***Pomacea canaliculata*** **transcriptome**

**Searching item**	**Numbers**
Total number of Unigene examined	135,121
Total size of examined Unigene (bp)	117,356,620
Total number of identified SSRs	16,717
Number of Unigene containing SSR	14,722
Number of Unigene containing more than 1 SSR	1,748
Number of SSRs present in compound formation	753
Di-nucleotide	10,554 (63.1%)
Tri-nucleotide	4,480 (26.8%)
Tetra-nucleotide	1,021 (6.10%)
Penta-nucleotide	321 (1.9%)
Hexa-nucleotide	341 (2.0%)

## Conclusions

The transcriptomes of the invasive golden apple snail (*P. canaliculata)* and the native mudsnail (*C. cahayensis*) were characterized using the Illumina next-generation sequencing technique. This allowed the identification of a number of the differentially expressed genes, some of which were found to be related specifically to environmental stress; for example, the CYP4 family of cytochrome P450s. These findings can contribute to a better understanding of pesticide metabolism and will provide valuable genetic data to facilitate future studies towards understanding the successful invasive and adaptive mechanism of *P. canaliculata*. In addition, the 16,717 EST-SSRs predicted in this study should provide a solid genetic basis for molecular markers development and aid in ecological studies pertaining to genetic variation in *P. canaliculata*.

## Methods

### Ethics statement

This study was approved by the Animal Care and Use committee of Aquatic Invasive Risk Assessment Center, Pearl River Fisheries Research Institute, Chinese Academy of Fishery Sciences.

### Sample collection, RNA extraction, and next generation sequencing

*P. canaliculata* (20–25 mm shell length; 25.23 ± 0.34 g; 10 individuals) and *C.cahayensis* (20.4–23.2 mm shell length; 22.43 ± 0.46 g; 10 individuals) were collected without the use of chemicals and grown in the Aquatic Invasive Risk Assessment Center, Pearl River Fisheries Research Institute, Chinese Academy of Fishery Sciences, Guangzhou, China. Tissues samples from the foot, muscle, liver, and kidney were rinsed separately with water pretreated by diethyl pyrocarbonate to cleanse the samples and inactivate RNases [32]. Total RNA of each sample was extracted using a Trizol Kit (Promega) according to the manufacturer’s instructions. RNA quality was assessed using a 2100 Bioanalyzer (Agilent Technologies, Santa Clara, CA) and RNase-free agarose gel electrophoresis, with the total RNA concentration measured using a 2100 Bioanalyzer. Equal amounts of RNA from each sampled tissue were combined for subsequent experiments and RNA purity was assessed at absorbance ratios of OD_260/280_ and OD_260/230_. RNA integrity was confirmed by 1% agarose gel electrophoresis.

### *De novo* assembly and gene annotation of Illumina reads

Transcriptome *de novo* assembly was carried out with the short-read assembly program Trinity [54]. The Trinity program has three independent modules: Inchworm, Chrysalis, and Butterfly. Inchworm assembled the RNA sequencing data into unique transcripts that we called Inchworm contigs; Chrysalis clustered the Inchworm contigs, then constructed complete de Bruijn graphs for each cluster and partitioned the full read set among these disjoint graphs; and Butterfly processed the individual graphs in parallel, tracing the paths based on reads and pair-end information, ultimately reporting full-length transcripts for alternatively spliced isoforms. After assembly, the TIGR Gene Indices clustering tools (TGICL) [55] were used to cluster and remove redundant transcripts. The remaining sequences after TGICL clustering were defined as unigenes. BLASTX searches (*E*-value < 10^−5^) were conducted to screen the unigenes against the Nr database (http://www.ncbi.nlm.nih.gov/), Swiss-Prot protein database (http://www.expasy. ch/sprot/), the KEGG pathway database (http://www.genome.jp/kegg/), and COG database (http://www.ncbi.nlm.nih.gov/cog/). High scoring alignments were used to determine the unigene sequence direction. When alignment results varied between databases, the results from the Nr database were preferentially selected, followed by the Swiss-Prot, KEGG and COG databases. When a unigene sequence did not match entries in any of these databases, ESTScan was used to predict the coding regions and determine sequence directionality.

### Functional annotation and differential expression analysis of unigenes

Unigene sequences were aligned to the protein databases (listed above) using BLASTX (E-value < 10^−5^) and to the nucleotide sequence database Nt (E-value < 10^−5^) using BLASTN to obtain both protein and functional annotation information. Based on the annotations in the protein databases, Blast2GO [56] was used to obtain GO annotations for the aligned unigene sequences and the Web Gene Ontology Annotation Plot (WEGO) software [57] was used to establish GO functional classifications for all unigenes. The unigenes were aligned to the COG database to predict and classify possible functions and the KEGG database was used [31] to obtain pathway annotations (E-value threshold 10^−5^). RPKM was used to calculate unigene expression levels, which eliminated the influence of gene length and sequencing level on the estimation of gene expression

### Analysis of genes related to pesticide

BLASTX searches against the Nr database (E-value < 10^−5^) were used to detect genes related to pesticide resistance. Sequences that returned redundant BLAST results or showed high sequence homology were eliminated and presumed to be allelic variants or different parts of the same gene. Thirty P450 gene sequences (Additional file 1: Table S1) with a range of bit-score values were identified and aligned using MUSCLE [58], and their phylogenetic relationships and genotype classifications were determined using MEGA 5 software [59]. The NJ method [60] was used to create phylogenetic trees, with positions containing alignment gaps or missing data eliminated via pairwise deletion. Tree branch strength was evaluated via a bootstrap analysis of 1000 replication trees.

### Development and detection of EST-SSR markers

MIcroSAtellite (MISA) (http://pgrc.ipk-gate-rsleben.de/misa/) was used for microsatellite mining. SSRs were considered to contain motifs of two to six nucleotides and a minimum of five contiguous repeat units. Based on the MISA results, Primer 6.0 was used with the default settings to design primer pairs that would generate PCR products ranging from 100 to 280 bp in length. A total of 100 pairs of primers were designed (Additional file 1: Table S1) and validated by PCR in six *P. canaliculata* samples, including Guangzhou 1–2 (GZ1-2), Hainan1-2 (HN1-2), and Shaoguang1-2 (SG1-2) that were collected from three major invasive regions in Guangdong Province, China. PCR amplification was carried out as follows: an initial denaturation at 94°C for 4 min; 33 cycles of 94°C for 1 min (denaturation), 50°C for 30 s (annealing), and 72°C for 45 s (extension); followed by a final extension at 72°C for 8 min. The PCR products were analyzed by electrophoresis on a 8.0% non-denaturing polyacrylamide gel and silver stained.

## Additional file

Additional file 1: Table S1. 60 P450-related gene sequences for phylogenetic tree in *Cipangopaludina cahayensis* and *Pomacea canaliculata.*
